# Ant-Plant Mutualism in *Mauritia flexuosa* Palm Peat Swamp Forests: A Study of Host and Epiphyte Diversity in Ant Gardens

**DOI:** 10.3390/insects15121011

**Published:** 2024-12-20

**Authors:** Yakov Quinteros-Gómez, Jehoshua Macedo-Bedoya, Abel Salinas-Inga, Flavia Anlas-Rosado, Victor Santos-Linares, Geancarlo Alarcon-Iman, Doris Gómez-Ticerán, Franco Angeles-Alvarez, Sergio Olórtegui-Chamolí, Julio Solis-Sarmiento, Enoc Jara-Peña, Octavio Monroy-Vilchis

**Affiliations:** 1 Laboratorio de Ecología Tropical y Análisis de Datos, Facultad de Ciencias Biológicas, Universidad Nacional Mayor de San Marcos (UNMSM), Lima 15081, Peru; yquinterosg@unmsm.edu.pe (Y.Q.-G.); jehoshua.macedo@unmsm.edu.pe (J.M.-B.); abel.salinas1@unmsm.edu.pe (A.S.-I.); flavia.anlas@unmsm.edu.pe (F.A.-R.); victor.santos2@unmsm.edu.pe (V.S.-L.); franco.angeles@unmsm.edu.pe (F.A.-A.); 2 Laboratorio de Entomología, Facultad de Ciencias Biológicas, Universidad Nacional Mayor de San Marcos (UNMSM), Lima 15081, Peru; 3 Departamento de Entomología, Museo de Historia Natural, Universidad Nacional Mayor de San Marcos (UNMSM), Lima 15081, Peru; geancarlo.alarcon@unmsm.edu.pe; 4 Facultad de Ciencias Matemáticas, Universidad Nacional Mayor de San Marcos (UNMSM), Lima 15081, Peru; dgomezt@unmsm.edu.pe; 5 Centro de Investigación, Conservación y Desarrollo Sostenible de Flora y Fauna Andino Amazónica del Perú—(CIFFA Perú), Moyobamba 22001, Peru; 72350633@continental.edu.pe; 6 Laboratorio de Genómica y Bioinformática Aplicada (Lab-Bio-Gen), Facultad de Ciencias Biológicas, Universidad Nacional Mayor de San Marcos (UNMSM), Lima 15081, Peru; 7 Laboratorio de Fitología Aplicada, Facultad de Ciencias Biológicas, Universidad Nacional Mayor de San Marcos (UNMSM), Lima 15081, Peru; ejarap@unmsm.edu.pe; 8 Centro Tlaxcala de Biología de la Conducta, Universidad Autónoma de Tlaxcala, Tlaxcala 90070, Mexico; 9 México y Ambiente ConCiencia A.C., Toluc 50170, Mexico

**Keywords:** ant-plant interaction, *Azteca*, *Camponotus*, hymenoptera, parabiosis, phorophytes

## Abstract

Mutualisms characterized by reciprocal benefits between species are a fundamental relationship of tropical ecosystems. Ant Gardens (AGs) represent an interesting ant-plant mutualism, involving specialized interactions between vascular epiphytes and ants. While this relationship has been extensively studied in various tropical regions, the available information on Peruvian ecosystems is limited. The objective of this study was to identify the ant and epiphyte species that constitute AGs. From February 2023 to January 2024, a study was conducted on two 50 × 10 m transects within the *Mauritia flexuosa* peat swamp forest, located within the Water Association Aguajal Renacal del Alto Mayo (ADECARAM) Tingana in San Martín, Peru. A total of 69 ant gardens were documented, comprising 18 phorophyte species, 19 epiphyte species, and three ant species. The results demonstrated that neither the height nor the diameter at breast height (DBH) of phorophytes exhibited a statistically significant correlation with the number of AGs per host. However, a positive correlation was observed between the length and width of the AGs and the number of ants per AG. The findings of this study contribute to the understanding of AG mutualism in Peruvian ecosystems.

## Simple Summary

Ant gardens (AGs) constitute a sophisticated example of mutualism between ants and plants (myrmecochory), characterized by intricate interspecific relationships with vascular epiphytes. Our main aim was to characterize the epiphytes and their associated ants in the Tingana Reserve in San Martín, situated at 800 m. a.s.l. This unique and humid ecosystem, distinguished by its altitude and microclimate, is home to a diverse array of plant species, including aguaje (*Mauritia flexuosa*). The area falls within the distribution range of Neotropical AGs, and our results highlight the ecological significance of ant specificity in seed dispersal among the epiphytes of the *Mauritia flexuosa* peat swamp forest in Peruvian ecosystems.

## 1. Introduction

Tropical rainforests are characterized by complex and dynamic systems, in which biotic interactions are the primary determinants of ecosystem function and structure [1]. Within these ecological interactions, the mutualistic association between epiphytic species and ants demonstrates highly specialized coevolutionary processes [2]. The ant gardens (AGs) represent a highly intricate form of ant-plant mutualism [3], leading to physiological and morphological adaptations that allow taxa to thrive in competitive environments [3,4]. The new information of these interactions contributes to enhancing the understanding of biodiversity and ecological processes in tropical forests.

In this complex system, ants select seeds from specific epiphytes, primarily guided by distinctive nutritional coatings or pheromones of the plant species. These seeds have morphological adaptations (specialized structures or appendages) that facilitate their transport and dispersal by the ants [5]. When germinated, the seeds not only transform the nest into an enriched microhabitat, but also moreover provide constant resources to the ants [6]. This interaction is a mutualism in which epiphytes receive protection from herbivory and continuous nutrients from the nitrogen-rich excretions of ants [7]. In addition, ants obtain benefits from the nectar and microhabitat provided by epiphytes [8]. It is worth noting that certain epiphytes, such as bromeliads, develop specialized structures capable of accumulating water, thus providing an optimal environment for ants [9].

Specific environmental factors of tropical ecosystems favor the presence of AGs. In fact, the extensive coverage and wide branching of tree vegetation in tropical forests acts as a filter for solar radiation at different levels of the canopy [10], allowing the development of epiphytic species associated with AG. These plants do not require permanent or direct exposure to light, but they do need moderate levels of light to carry out photosynthesis efficiently [11]. Furthermore, good air circulation is essential [12], as it not only facilitates transpiration and reduces the risk of fungal diseases in epiphytes, but also benefits ant colonies [13]. Rainfall and rain cycles influence ant activity patterns and epiphyte growth [14], with periods of lower rainfall coinciding with reduced periods of ant foraging, and periods of higher rainfall favoring expansion of AGs [15,16]. This balance culminates in a beneficial exchange, in return for the shelter, the ants carry out an active defense of the plant against herbivores, eradicating competing organisms and even removing nearby plant growth that could overshadow their host plant [17,18].

*Azteca*, *Camponotus*, and *Crematogaster* are genus ants and are distributed throughout the Neotropics, from Mexico to Brazil [19,20,21]. These ants typically construct their nests in shrubs and trees belonging to the families Lauraceae, Melastomataceae, Orchidaceae, Moraceae, Urticaceae, and Rubiaceae [22]. They exhibit a robust defense mechanism against external agents, effectively preventing herbivory [23,24], they participate in the dispersal of seeds of epiphytic species [25] and maintain a nutrient-rich substrate by incorporating organic waste such as vertebrate excreta [26].

Despite the crucial function of AGs, there remains a significant knowledge gap regarding the dynamics and diversity of epiphytes that comprise them in tropical zones, particularly in the Andean–Amazonian piedmont of Peru. This emphasizes the necessity for in-depth information in this area. The objective of this research was to determine the richness of ants, the composition of epiphytes, and the diversity of phorophytes in AGs in areas with different levels of human disturbance in the *Mauritia flexuosa* peat swamp forest of San Martín State, Peru. This research will provide important insights into mutualistic relationships in tropical ecosystems, which will support biodiversity conservation efforts and add to the limited knowledge of ant gardens.

## 2. Materials and Methods

### 2.1. Study Area

Alto Mayo Valley’s Andean–Amazonian piedmont is located between the yungas of the eastern Peruvian Andes and the low-lying seasonally flooded area of Central Huallaga [27]. This transitional area (20–30% slope) is characterized by a humid subtropical climate with rainfall concentrated during a single wet season between October and April [28]. The life zone is humid subtropical, mean temperature and annual precipitation are 22.8 °C and 1265 mm, respectively [29]. The soils of the Alto Mayo flooded forest are characterized by peat deposits accumulated since the Quaternary [30].

**Figure 1:**
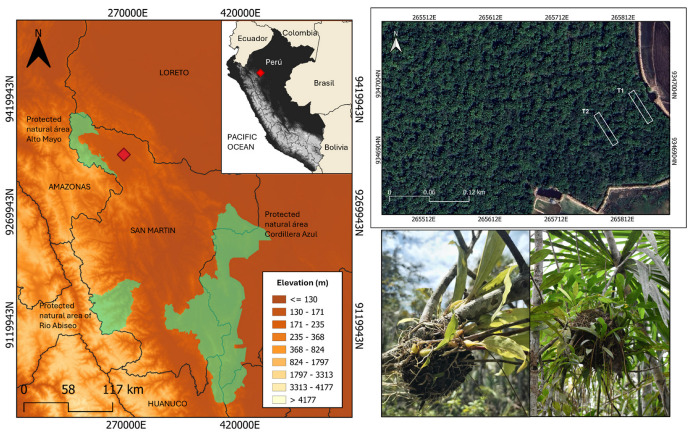
Study area in Andean–Amazonian piedmont (San Martín) and *Mauritia flexuosa* peat swamp forest, showing transect locations (up) and a typical garden tree.

The study area was in ADECARAM Tingana (Water Association Aguajal Renacal del Alto Mayo, ecotourism; 05°54′17.9″ S, 77°07′07.5″ W) and was carried out from February 2023 to January 2024 in a *Mauritia flexuosa* peat swamp forest with frequent anthropic activity (selective extraction of wood and vanilla) [31]. In these peat swamp forests, palms dominate the canopy (>20 m). Additionally, we find pioneer trees and shrubs (*Cecropia*, *Inga*, *Tococa*, *Miconia*) being colonized by epiphytic plants [32] in territories that were recently deforested and that have gaps in the canopy, in addition to the presence of peat and *Sphagnum* moss covering the ground. The recurrent floods caused by the Huascayacu and Avisado rivers, close to Tingana, cause recurrent flooding within the territories. This restricts the distribution of species that are unable to adapt to these conditions (Figure 1).

### 2.2. Field Sampling

Two 50 × 10 m transects were used for sampling, with all ant gardens identified. The first transect (T1) was situated at the forest edge close to agricultural lands, including rice, coffee, cocoa, and banana plantations. The second transect (T2) was positioned 50 m into the forest, parallel to the first. As a consequence of selective deforestation, the edges of the forest are becoming less visible in these territories.

For each phorophyte containing AGs, the following protocol was implemented:

(i) Photographic documentation of both the phorophytes and epiphytes;
(ii) Triplicate sampling of phorophytes and epiphytes;
(iii) Recording of dasometric variables for the phorophytes;
(iv) Measuring the length, width, and height of the AGs from the ground;
(v) Collecting samples of the associated ant species.

Moreover, 20 AGs were collected from both transects and transported to the laboratory for measurement and ant counting. Botanical nomenclature was based on W3-Tropicos (www.tropicos.org). The conservation status of recorded species was noted according to the Red List criteria and the Peruvian categorization of threatened flora species (Decreto Supremo N° 043–2006–AG). Ants associated with AGs were identified at the Entomology’s Department of the Natural History Museum at the Universidad Nacional Mayor de San Marcos, using keys from Longino [21,22,23,24,25,26,27,28,29,30,31,32,33], Mackay [34], and Feitosa and Dias [35]. Furthermore, the specimens were deposited at the Tropical Ecology and Data Analysis Laboratory at the Universidad Nacional Mayor de San Marcos.

### 2.3. Data Analysis

A one-way analysis of variance (ANOVA) was conducted to assess the significant effects of the two most common host species on epiphyte richness and the number of ant gardens (AGs) among the two most common hosts. A Pearson correlation was conducted to examine the relationship between the height and diameter at breast height (DBH) of the host trees and the number of AGs per host. The χ^2^ test was performed to determine if there were a significant difference for richness and abundance of epiphytes, and length and width of AGs between transects. A Pearson correlation was performed between the length and width of the AGs with 162 the abundance of ants per AGs. All statistical tests and correlation analyses were performed using the statistical software Statgraphics Centurion v.16.

Analysis of Similarities (ANOSIM) was performed using the Bray–Curtis distance (9999 permutations), considering the epiphytes composition species per transects and sorted the species composition using non-metric multidimensional scaling analysis (NMDS) with Bray–Curtis index. The data were analyzed using the gsankey library [36] to determine the preference of AG compositions over phorophytes in Tingana. The tripartite figure shows nodes representing species of epiphytes (left), ants (center) and host species (right). The NMDS and the tripartite figure were carried out with the Rstudio software version 2024.04.1+748 using the vegan packages developed by Oksanen et al. [37].

## 3. Results

### 3.1. Characterization of Phorophytes

**Figure 2:**
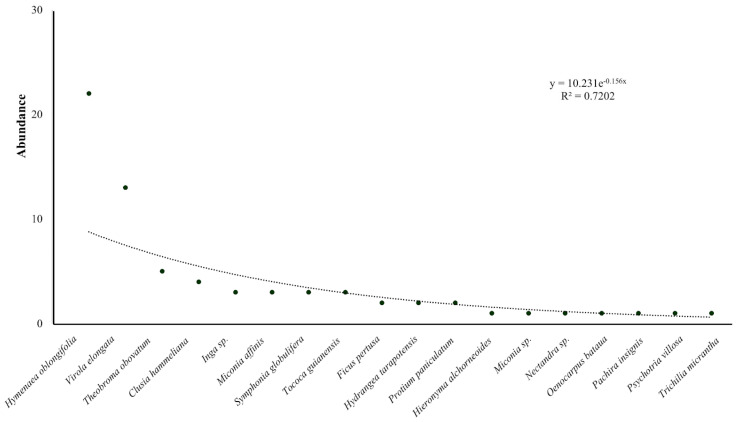
Dominance−diversity graph of phorophyte species in Tingana.

Eighty-nine AGs were found in 69 phorophytes belonging to 13 families, 17 genera, and 18 species (Table A1). *Hymenaea oblongifolia*, *Virola elongata*, and *Theobroma obovatum* were the most common phorophytes observed in Tingana, accounting for 64% of the observations (Figure 2). The botanical families Fabaceae, Myristicaceae, Melastomataceae, and Malvaceae were identified as the primary hosts for AG. The only palm species identified as a phorophyte was *Oenocarpus bataua*, with a single AG recorded at a height of 3 m.

A total of 78% of phorophytes had only one AG, while 17.4% had two ant gardens. *Hymenaea oblongifolia* and *Inga* sp. accounted for 50% of the phorophytes hosting two AGs. The only phorophytes with four AGs were *Theobroma obovatum* and *Tococa guianensis*. Richness (*T* = 1.21743; df = 1; *p* = 0.23207) and number of AGs per phorophyte (*T* = 0.687798; df = 1; *p* = 0.496387) did not show significant differences between the two most common phorophytes. Phorophyte height (*p* = 0.9463; *R* = 0.008) and DBH (*p* = 0.7244; *R* = −0.04) were not significantly related to the number of AGs per host.

### 3.2. Characterization of Epiphytic Species

**Figure 3:**
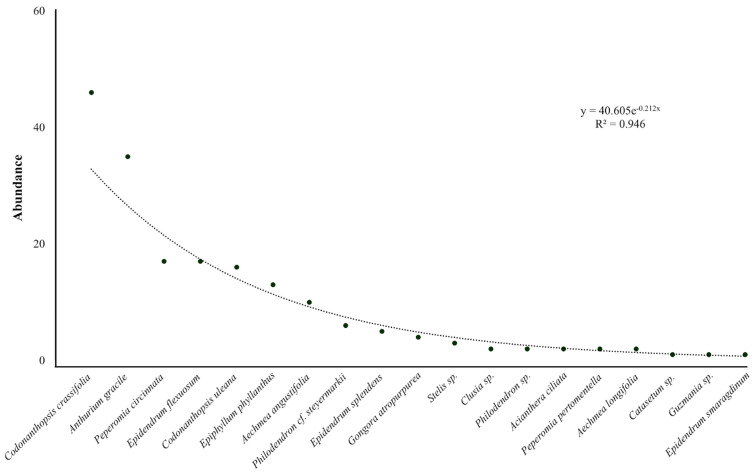
Dominance−diversity graph of epiphyte species in Tingana.

**Figure 4:**
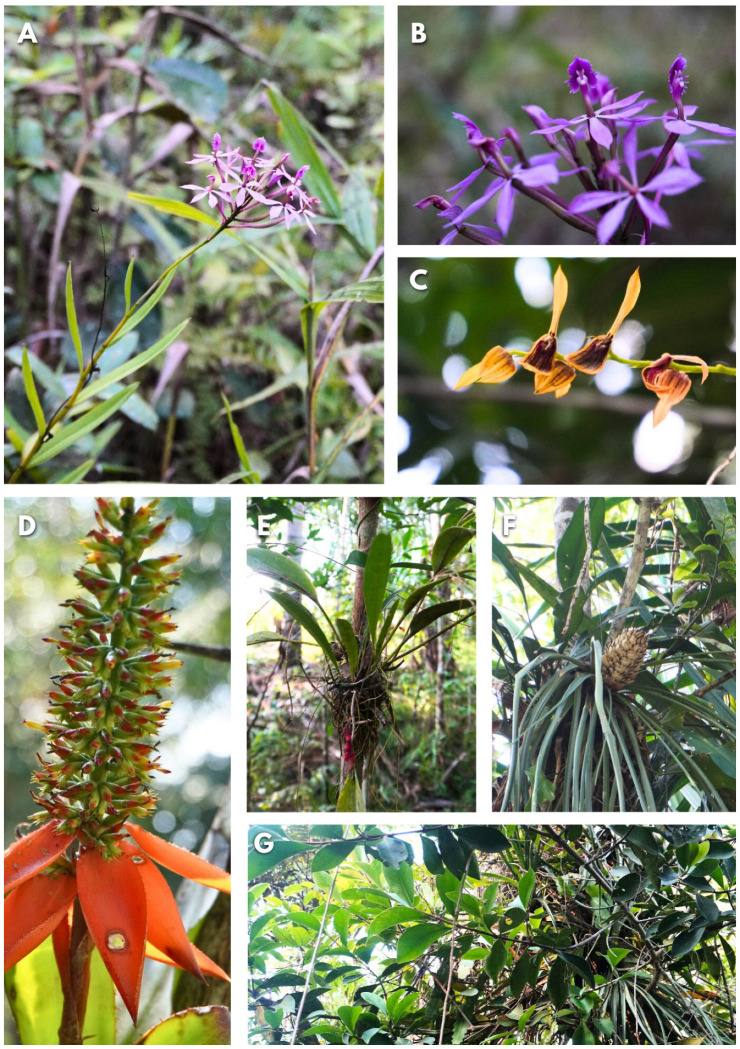
Epiphyte species reported in AGs: (**A**,**B**) *Epidendrum imatophyllum*, (**C**) *Acianthera lanceana*, (**D**) *Aechmea angustifolia*, (**E**) *Anthurium gracile*, (**F**) *Aechmea longifolia*, and (**G**) *Clusia* sp.

In the first and the second transect, 32 and 57 AGs, respectively, were recorded. A total of 180 epiphytic individuals were observed within the AGs, belonging to 19 species, 13 genera, and 7 families. The best represented families were *Orchidaceae* (seven species) and *Araceae* (three species), together accounting for 52.6% of the total richness. A total of 57.9% of the epiphytes were common to both transects. The most diverse genera were *Epidendrum*, *Aechmea*, *Codonanthopsis*, *Peperomia*, and *Philodendron*, and the most abundant species were *Codonanthopsis crassifolia*, *Anthurium gracile*, *Epiphyllum phyllanthus*, *Epidendrum* sp., *Peperomia circinnata*, and *Codonanthopsis uleana* (Figure 3 and Figure 4). The epiphytes *A. gracile*, *C. crassifolia*, and *C. uleana* were found in 62.5% of the AGs in the transects at the forest edge (T1) and in 94.7% of the AGs in the forest interior (T2), respectively.

The number of epiphytic species observed ranged from zero to five per AG. The abundance (*T* = −3.4677; df = 1; *p* < 0.001) and richness (*T* = −3.6200; df = 1; *p* = 0.000) of epiphyte species showed significant differences between the transects. On the contrary, the length (*T* =1.1037; df = 1; *p* = 0.2727) and width (*T* = −0.4761; df = 1; *p* = 0.6351) of the AGs showed no differences between transects but showed a positive correlation with the number of ants per AG (ength: *R* = 0.8579; *p* = 0.000; width: *R* = 0.9119; *p* < 0.001).

### 3.3. Characteristics of AGs

**Figure 5:**
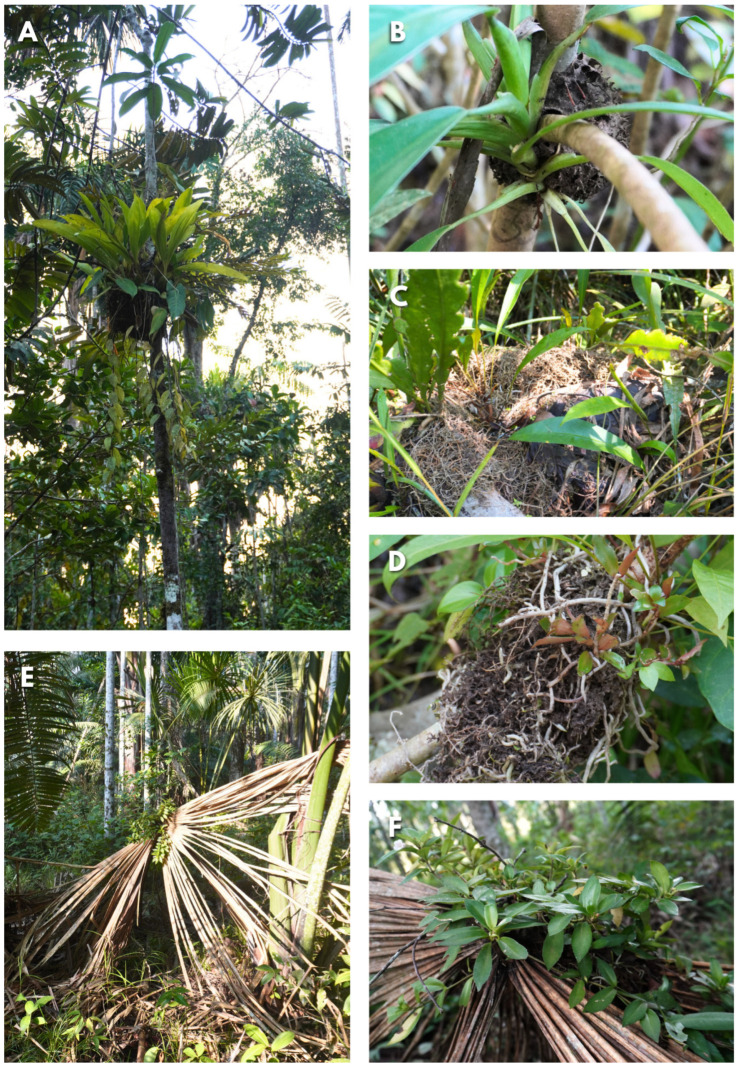
(**A**) AGs with high exposure, (**B**) AGs at intersection of phorophyte branches, (**C**) AGs collapsed by excess weight, (**D**) AGs show erosion, and (**E**,**F**) AGs on young leaves of *Mauritia flexuosa*.

A total of 62% of the AGs were observed in understory areas (less than 5 m in height) dominated by shrub vegetation and gaps in the canopy. In Tingana, the understory AGs had low light exposure, protected by the foliage of higher plants. In other cases, the AGs were exposed to more light intensity (greater than 10 m; 12%), with only the upper foliage of the phorophyte providing shade (Figure 5A) being the most abundant family Gesneriaceae (47%). Structurally, AGs are located where the main phorophyte axis crosses a secondary branch (Figure 5B). They are formed by the accumulation of leaves that decompose to form this assembly. However, not all trees or plants are conducive to the development of AGs. The stems of palm trees do not attract ants to form an AG.

In some cases, AGs grow to over 1 m long and wide, becoming extremely heavy for the developing shrub or tree to support; the main branch breaks, causing the AG to fall to the wet ground (Figure 5C), even submerging. The AG is quickly abandoned, and the epiphytes are deprived of light, water, and nutrients, and exposed to stress conditions leading to loss of AGs biomass (Figure 5D). The dominance of *Mauritia flexuosa* at the study site allowed the recording of many regeneration and juvenile individuals of this species. In fact, small AGs with *A. gracile* as the dominant epiphyte were observed on the underside of the leaves of young palms (maximum height: 3.5 m). The short lifespan of these leaves, generally around six months, limits the period during which they can support the colony (AG), with abandoned anthills observed on dry juvenile leaves of *M. flexuosa* (Figure 5E,F).

**Figure 6:**
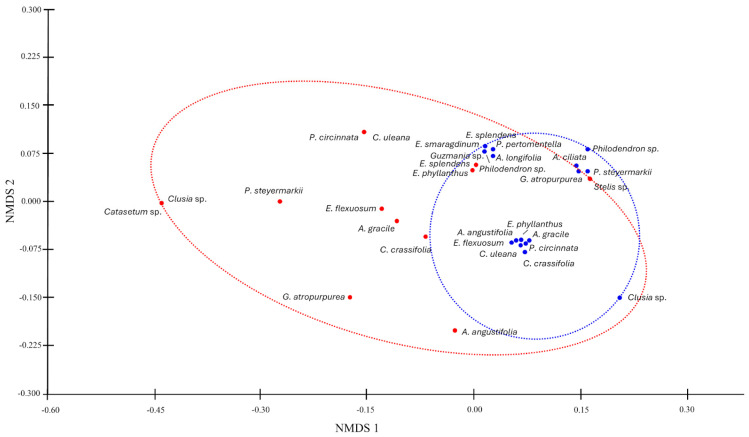
NMDS analysis of epiphyte species composition in AGs: comparing transects at forest edge (red ellipse) and interior forest (blue ellipse) of *Mauritia flexuosa* peat swamp forest in Tingana.

In the NMDS analysis, we observed that 12 epiphytic species were in both transects, forming a single assemblage with a stress level of 0.4076 (Figure 6; ANOSIM: *R* = 0.3316, *p* = 0.0001). This was evident from the proximity of the epiphytic species to one another, regardless of their respective transects. Most species in T2 were grouped within a blue ellipse, indicating a close relationship that suggests they may share common phorophytes. In contrast, the transect located at the edge of the agricultural perimeter (T1) exhibited isolated epiphytic species. For instance, *Hieronyma alchorneoides* served as a host for *Clusia* sp., *Philodendron* cf. *steyermarkii*, and *Catasetum* sp. These epiphytes are infrequently in the study area, which accounts for their considerable distance from their phorophyte.

### 3.4. Ants Diversity and Interactions

**Figure 7:**
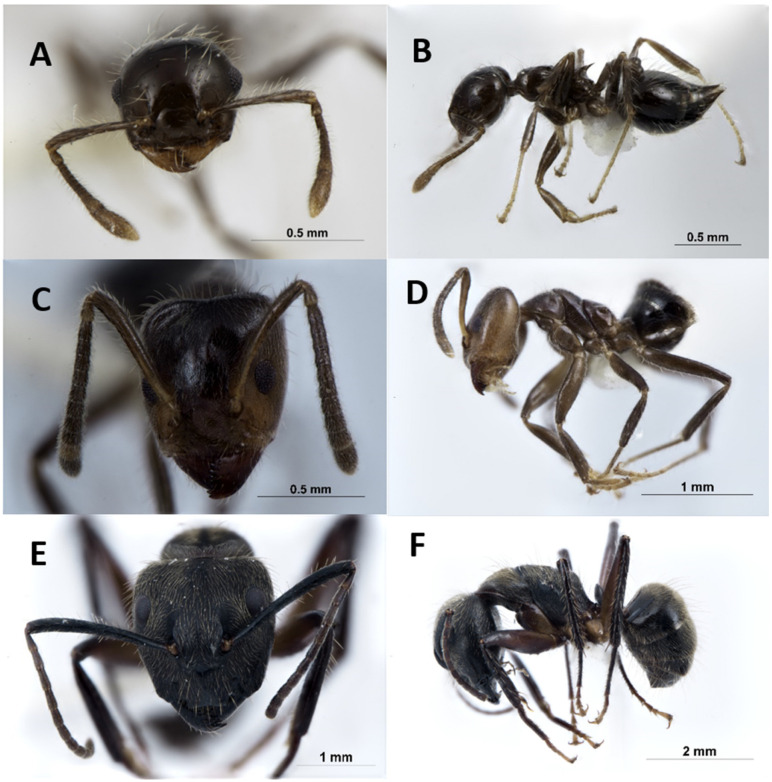
Ant species in the AGs of *Mauritia flexuosa* peat swamp forest: (**A**,**B**) *Crematogaster levior* (**C**,**D**), *Azteca instabilis*, and (**E**,**F**) *Camponotus femoratus*.

*Azteca instabilis* (Smith, 1862), *Camponotus femoratus* (Fabricius, 1804), and *Crematogaster levior* (Longino, 2003) were the ant species identified in the Tingana AGs studied (Figure 7). Among these, *A. instabilis* was more abundant, with 62.9% of the total records, while *C. femoratus* was the least abundant with 16.9%. We documented three AGs that were uninhabited (abandoned nests), 83 AGs hosting a single ant species, and three AGs with two ant species. The phorophyte with the highest ant diversity, three species, was *Hymenaea oblongifolia*.

*Clusia hammeliana* exclusively hosted *C. levior*, while *Protium paniculatum*, *Ficus pertusa*, and *Symphonia globulifera* were exclusively associated with *C. femoratus*. *T. guianensis* was the exclusive host for *A. instabilis* and no *C. levior* individuals were recorded on *Theobroma obovatum*. We encountered two ant species, *C. femoratus* and *C. levior*, that exhibited parabiosis within the phorophyte species *Miconia affinis* and *F. pertusa*, separately.

**Figure 8:**
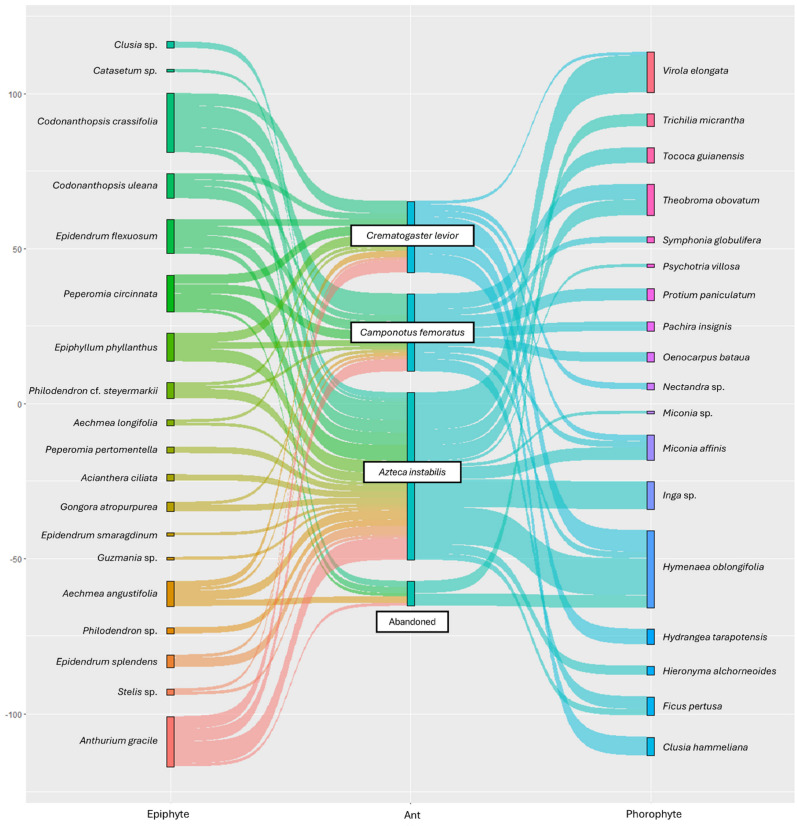
Tripartite graph showing the associations among phorophytes, ant species, and epiphytic species in AGs.

The complexity of the observed interactions between ants, their epiphytes, and phorophyte species is illustrated in a tripartite graph (Figure 8).

The tripartite graph illustrates the intricate web of interactions between epiphytes, ants, and phorophytes within the studied ecosystem. It is evident that ants such as *Crematogaster levior* and *Azteca instabilis* form associations with a considerable variety of epiphytes, which indicates a pivotal role for these species in the sustenance of ant gardens. Furthermore, the presence of abandoned gardens suggests that some epiphytes have lost their interactions with ants, which could potentially impact their development by leaving them without the protection and cleaning benefits that these provide. Regarding phorophytes, trees such as *Hymenanea oblongifolia* and *Virola elongata* exhibit extensive associations, acting as recurrent hosts for diverse ant and epiphyte species. These observations indicate the presence of a complex ecological structure, characterised by the coexistence of generalist and specialist ants, with potential influences from resource availability and tree characteristics on association dynamics (Figure 8).

### 3.5. Conservation Status

The phorophytes *F. pertusa*, *Hydrangea tarapotensis*, *Hymenaea oblongifolia*, *Miconia affinis*, *Miconia* sp., *Oenocarpus bataua*, *Pachira insignis*, *P. paniculatum*, *Psychotria villosa*, *S. globulifera*, *Theobroma obovatum*, *Tococa guianensis*, *Trichilia micrantha*, and *V. elongata*, as well as the epiphyte *E. phyllanthus*, are all considered as being of ‘Least Concern’ on the IUCN Red List of Threatened Species. The epiphytes *Epidendrum* sp., *Stelis* sp., and *Epidendrum splendens* are included in CITES Appendix II. In contrast, none of the ant species is threatened or listed in CITES.

## 4. Discussion

### 4.1. Phorophytes

Phorophytes play a crucial role in the development of ant gardens [38]. The structural characteristics of trees, such as diameter at breast height (DBH) greater than 10 cm, bark type, and crown architecture, significantly influence both epiphyte colonization and garden formation [39,40]. In this regard, few studies mention the hosts of AGs, and *Hymenaea oblongifolia*, *Virola elongata*, and *Theobroma obovatum* (dominant in Tingana) have not been previously reported. We attribute this to habitat specialization in Tingana [27], which is highly restrictive for those species not adapted to flooded ecosystems [32]. Common hosts in other Neotropical territories include *Miconia*, *Psychotria*, and the *Inga* genus [19,41].

The prevalence of pioneer species, adapted to high-light and disturbed environments, has been observed in Tingana and other disturbed regions [42]. Campos et al. [43] have demonstrated rapid colonization of these areas by ants and epiphytes. In contrast, ecosystems with lower disturbance levels and denser canopy cover tend to harbor different phorophyte species, such as *Tococa guianensis* and *Hymenaea oblongifolia*. In these environments, symbiotic relationships between plants and ants develop, indicating high ecological stability [44].

The ability of the *Hymenaea oblongifolia* to adapt to fluctuating flood conditions is crucial for maintaining mutualistic relationships between ants and epiphytes. Pioneer species (*Miconia* sp., *Miconia affinis*, *Symphonia globulifera*, and *Inga* sp.) can benefit from both post-disturbance high-light periods and flood-induced wet conditions [45].

Approximately 60% of myrmecophyte flora species are categorized as pioneers, exhibiting high light demand and rapid growth [46,47,48], as observed in Tingana. Myrmecophyte species predominantly occur in areas with human-induced disturbances, particularly near riparian vegetation or water bodies. This observation aligns with findings from the study area, as reported by Quinteros-Gómez et al. [32].

In the study area, trees and shrubs with higher DBH and height did not show more epiphytic colonization, as expected [49,50]. This is mainly because the area is in a secondary forest with selective anthropogenic activity [27,31], where individuals with DBH greater than 60 cm are rare. These are the individuals that regularly concentrate on the highest richness and abundance of epiphyte species [51,52].

The genera *Tococa* and *Hymenaea* are notable AG hosts across diverse ecological contexts [53]. *Tococa*, an ombrophilous genus, is better adapted to growth in less disturbed environments with limited sun exposure. Its leaves, often featuring extrafloral nectaries, attract ants that provide protection from herbivores, establishing mutualistic relationships in more closed and humid habitats [54,55]. Conversely, *Hymenaea* species adapt to varied light conditions, including more open and bright environments [56,57]. Although typically found in seasonally flooded or pre-montane forests [58], these species exhibit ecological plasticity that enables them to serve as phorophytes in areas with greater solar exposure. This facilitates ant and epiphyte interactions in more open environments (T1) [59], promoting ant proliferation that benefits both phorophyte and epiphyte protection [60].

### 4.2. Epiphytic Flora

The high diversity of epiphytic angiosperms in ant gardens (AGs) observed in this study parallels that of other Neotropical ecosystems, including those in Tabasco and Chiapas, Mexico [20], French Guiana [61,62], *Venezuelan rainforests* [63], and central Amazonian forests of Brazil [64]. The families Araceae, Bromeliaceae, Gesneriaceae, Piperaceae, and Orchidaceae show the highest diversity in these areas, suggesting a general pattern in AG structure and composition throughout the tropics, where mutualistic interactions between ants and epiphytes play a crucial role in shaping biodiversity [65]. Forest edge (T1) AGs exhibited lower epiphyte diversity, likely due to extreme conditions at the agricultural border [66]. In these conditions, *Codonanthopsis crassifolia* and *Anthurium gracile* predominate, distinguished by their ability to thrive in environments with significant humidity fluctuations and survive extended drought periods without growth impairment [67,68]. In contrast, the forest interior (T2), with its more stable microclimatic conditions, supports greater epiphyte diversity [69].

*Codonanthopsis crassifolia* exemplifies species that have developed specialized structures for ant interactions [70]. Its seeds feature a lipid- and amino acid-rich exocarp that attracts Crematogaster ants, which transport them to their nests [8]. The ants consume only the external covering, leaving the viable seed in the nest for germination [71]. *C. crassifolia* also possesses extrafloral nectaries that provide ant nutrition [72], and in return, ants protect the plant from herbivores and other threats [73]. This mutualism benefits both parties: the epiphyte gains dispersal and enhanced development, while ants receive lipids, nectar, and fruits [70].

*Peperomia pertomentella* represents another characteristic AG species. It demonstrates remarkable colonization capability across different phorophytes [74], establishing itself in diverse arboreal microhabitats within the Tingana forest [75].

These characteristics make them essential components of AGs, particularly in less disturbed forests [76]. While epiphytes can survive temporarily without ants following nest death [8], several factors facilitate their continued existence, primarily the phorophyte’s structural integrity and the availability of nitrogen-rich, organic substrates [77,78].

### 4.3. Ants and Mutualism

The genus *Azteca* was documented as the most abundant in Tingana ant gardens (AGs). *Azteca* is renowned for its aggressive defensive behavior towards hosts [79] and its capacity to establish nests across diverse tree and shrub families [22,80], as evidenced in Tingana with Fabaceae and Myristicaceae. This behavior enables *Azteca* to monopolize food resources and reduce herbivory damage, thereby promoting plant growth [81]. Conversely, *Crematogaster* species, which frequently engage in mutualistic associations [38], have been observed in association with *Hymenaea oblongifolia*, valued for its durable wood and aromatic resin. These plants attract ants through extrafloral nectary secretions, establishing a mutually beneficial relationship [6,34,82].

Significant geographical variation exists in ant-plant symbiotic associations [83]. For instance, *Tococa guianensis* establishes associations with *Azteca*, while *Camponotus femoratus* interacts mainly with the pioneer species *Symphonia globulifera*, *Theobroma obovatum* and *Miconia affinis* found in T1, where most anthropogenic activity takes place.

Resource availability significantly influences ant presence in tropical ecosystems [84]. Host plants provide diverse resources, including habitat, structural support, thermal regulation, climatic protection, and nutrition [85]. Ants utilize these resources for nest establishment and growth [4,86], constructing nests from fecal material, wood fragments, and leaves [87].

The *Miconia* and *Tococa* genera produces domatia, specialized ant shelters [8,88], conferring selective advantages in herbivore protection and competition [89]. Domatia size and plant characteristics influence ant occupancy [88]. In *Tococa guianensis*, no evidence suggests that *Azteca instabilis* receives nutritional rewards for protection [90]. These ants incorporate seeds into their nests, enabling germination and root development that creates supporting structures on host plant branches [91]. A family-level mutualism between Bromeliaceae (*Aechmea* and *Guzmania* in Tingana) and *Camponotus femoratus*, documented in French Guiana [92], represents one of the most intricate flowering plant mutualisms, being obligatory for Bromeliaceae [93,94] in partially shaded areas.

### 4.4. Ecological Importance

Ant gardens (AGs) exemplify a remarkable form of ant-plant mutualism and play a vital role in ecosystem conservation [95]. In these relationships, epiphytes gain three primary benefits: seed dispersal, herbivore protection, and nutrient access [94]. These conditions ensure optimal assemblage development and prevent epiphyte desiccation [19].

The ecological impact of AGs is significant, as they modify environments and maintain ecological balance [96]. The integration of diverse epiphytes into ant nests enhances biodiversity and creates microhabitats that attract various organisms [97]. Ant’s shape microclimatic conditions through their complex structures [95] and function as biocontrol agents, protecting plants from potential herbivores and competing species [89]. Notably, ants (particularly *Azteca instabilis*) play a crucial role in host plant defense by killing or deterring leaf-cutting ants and removing lepidopteran and beetle eggs from plant surfaces [23,55,98]. These interactions underscore the importance of understanding tropical ecosystem dynamics and how ants, serving as biological indicators, provide insights into ecosystem health and resilience in response to natural and anthropogenic disturbances [99].

AG establishment under favorable conditions depends on multiple factors, including relative humidity, temperature, soil type, vegetation, light intensity, and canopy cover [6,100]. These factors contribute to diverse microclimate formation that facilitates AG development. However, extremely dense canopies can impede light penetration, adversely affecting plant-ant interactions and inhibiting nest formation [101]. Reduced light availability can compromise the functionality of ants and epiphytes not specifically adapted to such conditions. Environmental conditions significantly influence ant behavior [102] and their epiphyte selection based on reciprocal benefits [73]. Ants contribute not only to AG construction but also to maintenance, regularly incorporating new materials and repairing substrates to maintain optimal conditions and prevent epiphyte desiccation.

AG longevity is often limited [103], influenced by excessive growth of certain epiphytes (including *A. longifolia*, *A. angustifolia*, *Philodendron* cf. *steyermarkii*, *C. uleana*, and *C. crassifolia*). When AGs become too heavy for developing shrubs or trees to support, branches break and fall, leading to ant nest abandonment.

## 5. Conclusions

In the Alto Mayo Valley’s Andean–Amazonian piedmont, 18 phorophyte species harbor ant gardens (AGs). Of these phorophytes, 78% supported a single AG, while 17.4% contained two AGs. The number of AGs per phorophyte ranged from 1 to 4, with single AGs being most common (78%). The AGs were associated with 19 epiphytic species. *Azteca instabilis*, *Camponotus femoratus*, and *Crematogaster levior* were the ant species identified in the AGs. Two ant species, *C. femoratus* and *C. levior*, exhibited parabiosis within separate phorophyte species, *Miconia affinis* and *Ficus pertusa*.

The differences in community composition between the two transects demonstrate how local factors, particularly light availability and microclimatic conditions, influence epiphyte distribution patterns.

## Data Availability

Data used in this study can be requested from the corresponding author via email: tavomonroyvilchis@gmail.com.

## Appendix A

**Table A1:** Data on phorophytes and their ant-gardens in a *Mauritia flexuosa* peat swamp forest in Tingana, Peru.

Phorophytes				Ant Gardens							
Family	Scientific Name	Height (m)	DBH (cm)	# AG	Ant Species		Epiphyte Species	Epiphyte Family	Height (cm)	Length (cm)	Width (cm)
Melastomataceae	*Miconia* sp.	2	5	1	*Azteca instabilis*		1	a	80	12	10
Clusiaceae	*Symphonia globulifera* L.f.	7	30	1	*Camponotus femoratus*		-	-	550	15	20
Myristicaceae	*Virola elongata* (Benth.) Warb.	9	43	1	*Azteca instabilis*		3	b	800	60	28
Phyllanthaceae	*Hieronyma alchorneoides* Allemão	5	20.5	1	*Azteca instabilis*		2, 8, 9	a, d, f	200	29	22
Myristicaceae	*Virola elongata* (Benth.) Warb.	5.5	21	1	*Azteca instabilis*		10	d	350	50	40
Melastomataceae	*Miconia affinis* DC.	3.5	9.5	2	*Camponotus femoratus*	*Crematogaster levior*	1, 2	a	200	12	8
					*Camponotus femoratus*	*Crematogaster levior*	1	a	100	7	8
Clusiaceae	*Symphonia globulifera* L.f.	4	13.7	1	*Camponotus femoratus*		5	d	200	7	8
Clusiaceae	*Symphonia globulifera* L.f.	6	15.5	1	*Camponotus femoratus*		5, 7	d, e	300	60	60
Melastomataceae	*Miconia affinis* DC.	2	5.5	1	*Azteca instabilis*		1, 2, 5	a, d	150	13	12
Fabaceae	*Hymenaea oblongifolia* Huber	8.5	24	1	*Azteca instabilis*		7, 12	a, e	350	35	98
Myristicaceae	*Virola elongata* (Benth.) Warb.	9.5	23.5	1	*Crematogaster levior*		10	d	850	16	15
Melastomataceae	*Miconia affinis* DC.	4.5	12.5	1	*Azteca instabilis*		1, 2, 11	a, d	100	15	12
Myristicaceae	*Virola elongata* (Benth.) Warb.	11	37.5	1	*Azteca instabilis*		10	d	1000	30	25
Fabaceae	*Hymenaea oblongifolia* Huber	10	30.5	1	*Azteca instabilis*		3, 11	b, d	700	30	22
Malvaceae	*Pachira insignis* (Sw.) Sw. exSavigny	8.5	31	1	*Camponotus femoratus*		3, 7, 16	b, d, e	700	60	55
Fabaceae	*Hymenaea oblongifolia* Huber	8	19.5	1	*Azteca instabilis*		7	e	600	11	10
Malvaceae	*Theobroma obovatum* Klotzsch ex Bernoulli	9	24.5	1	*Azteca instabilis*		1, 5	a, d	250	21	20
Myristicaceae	*Virola elongata* (Benth.) Warb.	8	21	1	*Azteca instabilis*		11	d	700	12	11
Fabaceae	*Hymenaea oblongifolia* Huber	18	60.5	1	*Azteca instabilis*		7	e	1300	40	35
Lauraceae	*Nectandra* sp.	3	9.8	1	*Crematogaster levior*		1, 7	a, e	200	25	20
Myristicaceae	*Virola elongata* (Benth.) Warb.	1.5	6.5	1	*Crematogaster levior*		-	-	200	7	8
Malvaceae	*Theobroma obovatum* Klotzsch ex Bernoulli	3.5	11	1	*Azteca instabilis*		5	d	250	11	7
Malvaceae	*Theobroma obovatum* Klotzsch ex Bernoulli	13	46.4	4	*Azteca instabilis*		3, 4, 5	b, c, d	250	25	18
					*Azteca instabilis*		7	e	400	5	7
					*Azteca instabilis*		1	a	700	12	10
					*Azteca instabilis*		1	a	1100	25	20
Malvaceae	*Theobroma obovatum* Klotzsch ex Bernoulli	8	22.6	1	*Camponotus femoratus*		3, 5	b, d	700	35	30
Malvaceae	*Theobroma obovatum* Klotzsch ex Bernoulli	9	22.4	2	*Camponotus femoratus*		7, 11	d, e	300	20	15
					*Camponotus femoratus*		6	a	400	21	18
Moraceae	*Ficus pertusa* L. f.	2.5	7	1	*Camponotus femoratus*	*Azteca instabilis*	7, 13	e, g	100	20	17
Fabaceae	*Hymenaea oblongifolia* Huber	12	46.7	1	*Azteca instabilis*		7	e	1100	45	32
Fabaceae	*Hymenaea oblongifolia* Huber	11	25	1	*Azteca instabilis*		1, 7, 11	a, d, e	650	30	23
Fabaceae	*Hymenaea oblongifolia* Huber	11	16	1	*Azteca instabilis*		7	e	1000	8	8
Fabaceae	*Hymenaea oblongifolia* Huber	14	35.2	1	*Azteca instabilis*		7, 13	e, g	400	65	50
Fabaceae	*Hymenaea oblongifolia* Huber	11	29.5	3	*Azteca instabilis*		1, 6, 7	a, e	700	40	32
					*Azteca instabilis*		1	a	600	12	17
					*Azteca instabilis*		6	e	900	15	15
Burseraceae	*Protium paniculatum* Engl.	9	29	1	*Camponotus femoratus*		5, 7	d, e	700	25	14
Rubiaceae	*Psychotria villosa* Ruiz & Pav.	2.5	5.8	1	*Azteca instabilis*		7	e	200	9	4
Burseraceae	*Protium paniculatum* Engl.	8	19	1	*Camponotus femoratus*		1, 4, 7	a, c, e	300	60	50
Arecaceae	*Oenocarpus bataua* Mart.	8	16.3	1	*Camponotus femoratus*		1, 4, 7	a, c, e	300	50	50
Melastomataceae	*Tococa guianensis* Aubl.	3.5	8	4	*Azteca instabilis*		1, 6	a, e	100	9	7
					*Azteca instabilis*		6	e	100	12	11
					*Azteca instabilis*		6	e	200	4	4
					*Azteca instabilis*		1	a	250	30	70
Meliaceae	*Trichilia micrantha* Benth.	9	18	2	Abandoned		5, 7, 13	d, e, g	200	18	12
					Abandoned		1	a	500	15	11
Fabaceae	*Inga* sp.	10	25.2	1	*Azteca instabilis*		1, 4, 7, 12, 13	a, c, e, g	700	80	37
Melastomataceae	*Tococa guianensis* Aubl.	3.5	9.4	1	*Azteca instabilis*		3, 9	b, f	200	18	17
Clusiaceae	*Clusia hammeliana* Pipoly	18	34	1	*Crematogaster levior*		3, 5	b, d	600	80	70
Fabaceae	*Hymenaea oblongifolia* Huber	4	12.8	2	*Azteca instabilis*		1, 14, 15	a, d, g	200	8	6
					*Azteca instabilis*		1	a	300	18	14
Melastomataceae	*Tococa guianensis* Aubl.	2	4.6	1	*Azteca instabilis*		4, 6	c, e	150	7	7
Fabaceae	*Hymenaea oblongifolia* Huber	12	35.2	1	Abandoned		4, 5, 7, 13	c, d, e, g	400	113	97
Fabaceae	*Hymenaea oblongifolia* Huber	4	8.6	1	*Azteca instabilis*		4, 5, 7	c, d, e	200	14	7
Fabaceae	*Hymenaea oblongifolia* Huber	16	44.5	1	*Camponotus femoratus*		1, 7	a, e	1000	60	43
Fabaceae	*Hymenaea oblongifolia* Huber	3	9.5	1	*Azteca instabilis*		4, 7, 13	c, e, g	300	18	15
Fabaceae	*Inga* sp.	4	10.5	2	*Azteca instabilis*		1, 4, 6, 7	a, c, e	200	13	9
					*Azteca instabilis*		1, 16	a, d	300	6	6
Hydrangeaceae	*Hydrangea tarapotensis* Briq.	3	14	1	*Crematogaster levior*		6, 13	e, g	300	40	37
Fabaceae	*Hymenaea oblongifolia* Huber	4	4.8	1	*Azteca instabilis*		7	e	300	26	23
Fabaceae	*Hymenaea oblongifolia* Huber	3	27.9	2	*Crematogaster levior*		1, 7, 19	a, e, g	250	12	9
					*Crematogaster levior*		1	a	300	10	8
Fabaceae	*Hymenaea oblongifolia* Huber	21	66.9	2	*Crematogaster levior*		5, 6	d, e	1400	21	18
					*Crematogaster levior*		6	e	1100	40	32
Hydrangeaceae	*Hydrangea tarapotensis* Briq.	10	19.8	2	*Crematogaster levior*		3, 4, 7	b, c, e	400	100	80
					*Crematogaster levior*		7	e	600	16	14
Myristicaceae	*Virola elongata* (Benth.) Warb.	9	24.6	2	*Azteca instabilis*		3, 4, 7	b, c, e	400	40	28
					*Azteca instabilis*		7, 17, 19	d, e, g	800	23	20
Fabaceae	*Hymenaea oblongifolia* Huber	20	72	1	*Crematogaster levior*		3, 6	b, e	1500	135	118
Fabaceae	*Hymenaea oblongifolia* Huber	10	53	1	*Crematogaster levior*		1, 4, 6	a, c, e	800	45	30
Fabaceae	*Hymenaea oblongifolia* Huber	13	30.7	2	*Azteca instabilis*		3, 7, 13	b, e, g	800	12	14
					*Azteca instabilis*		4, 7	c, e	700	25	19
Clusiaceae	*Clusia hammeliana* Pipoly	24	69.7	1	*Crematogaster levior*		3, 5, 13	b, d, g	2200	18	12
Clusiaceae	*Clusia hammeliana* Pipoly	4.5	69.7	1	*Crematogaster levior*		4, 5, 7	c, d, e	150	12	8.5
Myristicaceae	*Virola elongata* (Benth.) Warb.	12	25.5	2	*Azteca instabilis*		5, 7, 16	d, e	600	25	18
					*Azteca instabilis*		1	a	700	20	13
Myristicaceae	*Virola elongata* (Benth.) Warb.	6	11.5	1	*Azteca instabilis*		1, 7	a, e	400	28	14
Fabaceae	*Hymenaea oblongifolia* Huber	18	14.4	1	*Azteca instabilis*		7	e	400	45	33
Fabaceae	*Hymenaea oblongifolia* Huber	18	56.8	1	*Azteca instabilis*		3, 7, 18	b, e	1200	60	52
Clusiaceae	*Clusia hammeliana* Pipoly	18	61.8	1	*Crematogaster levior*		1, 5, 7	a, d, e	300	65	52
Myristicaceae	*Virola elongata* (Benth.) Warb.	6	17.5	1	*Azteca instabilis*		1, 2	a	200	10	8
Myristicaceae	*Virola elongata* (Benth.) Warb.	4	18.5	1	*Azteca instabilis*		7	e	250	15	11
Fabaceae	*Inga* sp.	4	16	2	*Azteca instabilis*		1, 4, 7, 18	a, c, e	200	16	13
					*Azteca instabilis*		6, 7, 17	d, e	300	14	11
Myristicaceae	*Virola elongata* (Benth.) Warb.	5	14	1	*Azteca instabilis*		1, 7, 16	a, d, e	400	10	7
Myristicaceae	*Virola elongata* (Benth.) Warb.	2.5	12.5	1	*Azteca instabilis*		1, 4, 6, 7	a, c, e	200	18	16
Moraceae	*Ficus pertusa* L. f.	20	49	1	*Camponotus femoratus*		4, 6, 7	c, e	100	20	20

Epiphyte species: (1) *Anthurium gracile* (Rudge) Schott, (2) *Philodendron* cf. *steyermarkii* G.S. Bunting, (3) *Epiphyllum phyllanthus* (L.) Haw, (4) *Peperomia circinnata* Link, (5) *Epidendrum flexuosum* G.Mey., (6) *Codonanthopsis uleana* (Fritsch) Chautems & Mat. Perret, (7) *Codonanthopsis crassifolia* (H.Focke) Chautems & Mat.Perret, (8) *Catasetum* sp., (9) *Clusia* sp., (10) *Stelis* sp., (11) *Epidendrum splendens* Schltr., (12) *Philodendron* sp., (13) *Aechmea angustifolia* Poepp. & Endl., (14) *Guzmania* sp., (15) *Epidendrum smaragdinum* Lindl., (16) *Gongora atropurpurea* Hook., (17) *Acianthera ciliata* (Knowles & Westc.) F.Barros & L.R.S.Guim., (18) *Peperomia pertomentella* Trel., and (19) *Aechmea longifolia* (Rudge) L.B.Sm. & M.A.Spencer. Botanic family of epiphyte species: (a) Araceae, (b) Cactaceae, (c) Piperaceae, (d) Orchidaceae, (e) Gesneriaceae, (f) Clusiaceae, and (g) Bromeliaceae.
